# Factors affecting current khat chewing among male adults 15–59 years in Ethiopia, 2016: a multi-level analysis from Ethiopian Demographic Health Survey

**DOI:** 10.1186/s12888-020-2434-7

**Published:** 2020-01-14

**Authors:** Temesgen Yihunie Akalu, Adhanom Gebreegziabher Baraki, Haileab Fekadu Wolde, Ayenew Molla Lakew, Kedir Abdela Gonete

**Affiliations:** 1 Department of Epidemiology and Biostatistics, Institute of Public Health, College of Medicine and Health Sciences, University of Gondar, Gondar, Ethiopia; 2 Department of Human Nutrition, Institute of Public Health, College of Medicine and Health Sciences, University of Gondar, Gondar, Ethiopia

**Keywords:** Khat, Ethiopia, EDHS, Multilevel

## Abstract

**Background:**

Regular khat chewing causes gingivitis, tooth loss, gastric disorders, cardiac complications, male impotence, sleeplessness, and several mental health problems. Based on the Ethiopian Demographic and Health Survey (EDHS) 2016, 12% of women and 27% of men have reported having ever chewed khat. Even though khat addiction is a major public health problem, studies that consider both individual level and community level factors are limited. Therefore, this study aimed to determine the prevalence and factors affecting current khat chewing among male adults in Ethiopia.

**Methods:**

Data from EDHS, a community-based cross-sectional study conducted from January 18, 2016, to June 27, 2016, was used. A multistage stratified cluster sampling technique was used to select participants. Both descriptive and analytical statistics were done. Bi-variable and multivariable multilevel logistic regression analyses were performed to determine factors affecting current khat chewing. Adjusted Odds ratio (AOR) with 95% Confidence Interval (CI) for variables with *P*-value < 0.05 was used as a measure of association.

**Result:**

A total of 12,595 male adults were included. The prevalence of current khat chewing was 23.61% (95% CI: 22.87, 24.36). Age 20–24 years; (AOR = 2.68, 95% CI: 2.02, 3.56), being uneducated (AOR = 1.62, 95% CI: 1.10, 2.39), professional/technical/managerial job (AOR = 3.59, 95% CI: 2.18, 5.91), Muslim religion (AOR = 18.30, 95% CI: 13.54, 24.74), poorest wealth index (AOR = 0.67, 95% CI: 0.51, 0.89), being divorced (AOR = 0.38, 95% CI: 0.21, 0.69), history of alcohol drinking in the last 30 days (AOR = 2.15, 95% CI: 1.69, 2.73), and history of cigarette smoking in the last 30 days (AOR = 14.92, 95% CI: 10.88, 20.47), and Amhara region (AOR = 0.07, 95% CI: 0.04, 0.14) were significantly associated with khat chewing.

**Conclusion:**

Khat chewing remains high in Ethiopia with certain regional variations. The uneducated, older age, Alcohol and cigarette users, Muslims, and professional workers were at higher risk of khat chewing whereas the poorest wealth index and being divorced reduced its risk. Policymakers should consider a multi-faceted policy approach that accounts for regional variation and the identified risk factors to alleviate the problem.

## Background

Khat, native to Ethiopia, and the southern Arabian Peninsula is an evergreen shrub cultivated as a bush or small tree [1]. Its young buds and tender leaves (the main utilized part) contain amphetamine-like psychoactive substances, which produce euphoria and stimulation [2]. People chew khat for a variety of reasons, these include entertainment, as part of social events, to spend time, as a culture and some also use it to stay active when studying [3].

Regular khat chewing: causes gingivitis, tooth loss, gastric disorders, cardiac complications, male impotence, sleeplessness, and several mental health problems [4–7]. A study from Ethiopia suggests that khat chewing resulted in poor lung function and oxygen saturation [8], risky sexual behavior [9, 10], and psychosis [11]. In addition, concurrent use of khat with alcohol and cigarette smoking was highly associated with sleep disturbance [12].

High prevalence of khat chewing has been recorded in Yemen and the Jazan region of Saudi Arabia. According to global synthetic drug assessment in 2014, the prevalence of khat chewing among people aged 16 and above in Yemen was 52% [13]. In the last several years, khat has been increasingly transported from East Africa including (Ethiopia, Kenya, and Uganda) and the Arabian Peninsula (Yemen) to other regions [14]. A large amount of khat is smuggled from Ethiopia and Kenya to the United Kingdom and the Netherlands by plane, where until recently it has not been under national control in either of the countries [15].

Based on the Ethiopian Demographic and Health Survey (EDHS) 2016, 12% of women and 27% of men report having ever chewed khat [16]. Khat consumption increases with age and peaks at age 30–34 among both women (15%) and men (34%) [17].

The major predisposing factors for khat chewing include; family member who had history of khat chewing [18, 19], need of concentration and relaxation [13, 20], smoking cigarette, alcohol drinking [6, 21], religious practice [13, 22], economical status [5], peer pressure [5, 19, 23], place of residence [19], and increased workload [22].

Globally, in different countries khat and its consumption has been regulated by the Government [24]. In Ethiopia, especially in the Amhara region, the regional government imposed policy and increased taxation. But the problem remains high and evidence that shows the determinants of khat chewing especially that entertain both individual level and community level factors are limited. Multilevel models can be used to draw individual-level inferences, but inferences can also be made regarding group-to-group variation, including whether it exists in the data, and the extent to which it is accounted for by group and individual-level characteristics [25]. Therefore, this study was aimed to assess the predisposing factors of khat chewing among male adults in Ethiopia.

## Methods

### Study design and setting

Data from EDHS 2016 was used. EDHS 2016 is a community-based cross-sectional study conducted from January 18, 2016, to June 27, 2016. Ethiopia is situated in the Horn of Africa between 3 and 15 degrees north latitude and 33 and 48 degrees east longitude. Administratively Ethiopia has nine regional states. Namely: Tigray, Afar, Amhara, Oromia, Somali, Benishangul-Gumuz, Southern Nations Nationalities and peoples Region (SNNPR), Gambella, and Harari. In addition, it has two city administrations, Addis Ababa and Dire-Dawa. More than 82% of the country’s total population lives in the regional states of Amhara, Oromia, and SNNPR [11].

### Population, sample size, and sampling technique

All men from 15 to 59 years in Ethiopia were the source population. Whereas, all men age 15–59 years in the selected enumeration areas (EAs) were the study population. All men age 15–59 who were the members of the selected households and those who spent the night before the survey in the selected households were included in the study.

The 2016 EDHS used a two-stage stratified cluster sampling technique with regions and residence as strata were used. Primarily, all nine regions were stratified into urban and rural clusters. A total of 654 EAs (202 urban and 443 rural clusters) were selected proportional to EA size. In the second stage of selection, a fixed number of 28 households per cluster were selected from the newly updated listing of households. Altogether, 16,650 households and 12,688 men aged 15–59 years were interviewed in the survey.

### Study variables

Current khat chewing status, the outcome variable in this study was defined khat chewing within 30 days prior to the data collection period. The independent variables were grossly classified into socio-demographic and economic factors: age, religion, marital status, educational status, place of residence, region, and behavioral factors: alcohol drinking and smoking, and source of information: reading news-paper, reading magazines, and watching television.

Wealth ranking was grossly categorized into 5 major quintiles based on household assets as lowest (poorest), second (poorer), middle (middle), fourth (richer), and highest (richest). Substance use: was defined as the use of one or more of the substance like khat, Alcohol, using any type of tobacco (shisha or Gaya).

### Data collection tool and procedure

The data was taken from EDHS which is collected by trained data collectors using standardized, structured, and pre-tested questionnaires. The questionnaire, based on the DHS Program’s standard Demographic and Health Survey questionnaires, were adapted to reflect the population and health issues relevant to Ethiopia. The input was solicited from various stakeholders representing government ministries and agencies, nongovernmental organizations, and international donors. After all, questionnaires were finalized in English, they were translated into local languages. Raw data collected from all part of the country on Men whose age was between 15 and 59 were extracted for the analysis.

### Data processing and analysis

STATA version 14 was used for data analysis. Both descriptive and analytical analysis was done and presented using tables and texts. Bi-variable and multivariable multi-level logistic regressions were performed to determine the existing association among individual and community factors. Initially, a bi-variable analysis was performed and variables with a *p*-value of less than 0.2 were used for further analysis in the multivariable multilevel logistic regression. At the same time, Crude Odds Ratio (COR) and Adjusted Odds Ratio (AOR) with their corresponding confidence intervals (CI) were determined for the bi-variate and multi-variable analysis, respectively. The presence or absence of multi-collinearity was checked using the Variance Inflation Factor (VIF). Finally, *p*-value < 0.05 was used to declare the significance of association in the multi-variable model. The need for multi-level multivariable analysis which considers community-level factors was tested using the intra-class correlation coefficient (ICC) and models were compared using the deviance tests. The model with the lowest deviance was selected as the best-fitted model. In addition, sensitivity and specificity tests were checked. The presence and absence of univariate and multivariate outliers were checked.

## Result

### Baseline characteristics of study participants

A total of 12,595 participants were included in the study. The mean age of the respondents was 30.7 (SD = 11.5). One-fifths, 2572 (20.4%) of participants were in the age of 15–19 years. The majority of the participants, 10,099 (80.2%) were from rural and 5876 (46.7%) had primary education. More than half, 6968 (55.3%), of participants, were married (Table 1).

**Table 1:** Socio-demographic and behavioral factors of adult men 15–59 years in Ethiopia

Variable	Frequency	Percentage
Age in 5 year groups		
15–19	2572	20.4
20–24	1880	14.9
25–29	1975	15.7
30–34	1614	12.8
35–39	1371	10.9
40–44	1188	9.4
45–49	933	6.4
50–54	575	4.6
55–59	487	3.9
Residence		
Urban	2496	19.8
Rural	10,099	80.2
Usual resident or visitor		
Usual resident	12,316	97.78
Visitor	279	2.22
Region		
Tigray	795	6.3
Afar	82	0.65
Amhara	3206	25.5
Oromia	4714	37.43
Somali	326	2.6
Benishangul	123	0.98
SNNPR	2586	20.5
Gambella	37	0.29
Harari	31	0.25
Dire-Dawa	72	0.57
Addis Ababa	621	4.9
Educational level		
No education	3773	29.96
Primary education	5876	46.7
Secondary education	1846	14.7
Higher education	1099	8.7
Occupation		
Not working	944	9.5
Professional/technical/managerial	630	5
Clerical	97	0.8
Sales	677	5.4
Agriculture – employee	8336	66.2
Services	210	1.7
Skilled manual	824	6.5
Unskilled manual	279	2.2
Others	598	4.8
Religion		
Orthodox	5677	45.1
Catholic	90	0.7
Protestant	2745	21.8
Muslin	3916	31.1
Traditional + other	166	1.3
Sex of household head		
Male	11,034	87.6
Female	1561	12.4
Wealth Index		
Poorest	1979	15.7
Poorer	2294	18.2
Middle	2427	19.3
Richer	2712	21.5
Richest	3183	25.3
Current marital status		
Never in union	4890	38.8
Married	6968	55.3
Living with partner	421	3.3
Widowed	44	0.4
Divorced	226	1.8
No longer living together/separated	46	0.4
Media Exposure		
Yes	8154	64.7
No	4441	35.3
Alcohol drinking in the last 30 days		
Yes	5428	43.1
No	7167	56.9
Smoking cigarette in the last 30 days		
Yes	655	5.2
No	11,940	94.8

### Prevalence of current khat chewing

A total number of 2974 participants chewed khat in the last 30 days prior to the survey. This makes the prevalence of current khat chewing 23.61% (95% CI: 22.87, 24.36).

### The multilevel mixed-effects binary logistic regression model

The need for the multilevel model was confirmed by ICC of 66% with 95% CI (61.8, 70%). We have also compared the four models (Null model, Individual-level, community level and a model with both individual and community-level factors) and we have found the last model with the lowest deviance to be the best-fitted model (Table 2).

**Table 2:** Model comparison for identifying factors affecting khat chewing among male adults 15–59 years in Ethiopia, 2016

Model	Deviance
Model I (Null Model)	3869.58
Model II (individual level factors)	2944.39
Model III (community-level factors)	3745.34
Model IV (both individual and community-level factors)	2858.58

### Factors affecting current khat chewing among male adults in Ethiopia

In the bi-variable multilevel logistic regression analysis; age, sex of household head, residence, educational status, occupation, religion, wealth Index, current marital status, alcohol drinking history in the last 30 days, cigarette smoking history in the last 30 days, and region were associated factors with khat chewing with a *p*-value < 0.2. In the multivariable analysis Age, educational status, occupation, religion, wealth index, current marital status, alcohol drinking history in the last 30 days, cigarette smoking history in the last 30 days, and region were significantly associated with current khat chewing at a p-value < 0.05.

The odds of khat chewing among older adults were higher than that of teenagers aged between 15 and 19 years. When compared to adults who had higher educational status those who had no education (AOR = 1.62, 95% CI: 1.10, 2.39) and primary education (AOR = 1.85, 95% CI: .30, 2.62) had higher odds of chewing khat. When compared to participants who have no job those with jobs have higher odds of Khat chewing. Muslim study participants had higher odds of chat chewing (AOR = 18.30, 95% CI: 13.54, 24.74) when compared to Orthodox Christians whereas Catholics (AOR = 0.16, 95% CI: 0.05, 0.48) and Protestants (AOR = 0.20, 95% CI: 0.13, 0.31) had lower odds of khat chewing. Participants who have poorer wealth index had 33% lower odds of Khat chewing (AOR = 0.67, 95% CI: 0.51, 0.89) when compared to the poorest. The odds of being current khat chewer among divorced men were reduced by 62% (AOR = 0.38, 95% CI: 0.21, 0.69) as compared to singles. Participants who drink alcohol in the last 30 days had 2.15 (AOR = 2.15, 95% CI: 1.69, 2.73) times higher odds of being current khat chewer than their counterparts likewise those who smoke cigarette in the last 30 days had 14.92 (AOR = 14.92, 95% CI: 10.88, 20.47) times higher odds of being current khat chewer than those who do not. The odds of khat chewing had also a significant difference among regions of Ethiopia (Table 3).

**Table 3:** Multivariable logistic regression analysis among adult males in Ethiopia, 2016

Variable	Khat chewing		COR(95% CI)	AOR(95% CI)	AOR(95% CI)
	Yes	No			
Individual-level factors					
Age					
15–19	331	2241	1.00	1.00	1.00
20–24	394	1486	3.0 (2.35, 3.83)	2.66 (2.00, 3.52)	2.68 (2.02, 3.56)*
25–29	577	1398	6.11 (4.84, 7.72)	6.27 (4.58, 8.59)	6.24 (4.55, 8.56)*
30–34	500	1114	6.72 (5.26, 8.57)	6.74 (4.75, 9.56)	6.59 (4.64, 9.36)*
35–39	375	996	5.77 (4.43, 7.51)	5.19 (3.56, 7.57)	4.92 (3.37, 7.18)*
40–44	317	871	5.94 (4.57, 7.73)	5.05 (3.45, 7.42)	4.99 (3.40, 7.34)*
45–49	222	711	4.01 (2.98, 5.39)	2.91 (1.92, 4.39)	2.89 (1.91, 4.37)*
50–54	141	434	4.60 (3.25, 6.49)	3.91 (2.45, 6.24)	3.81 (2.38, 6.10)*
55–59	116	371	4.49 (3.15, 6.40)	4.74 (2.94, 7.65)	4.66 (2.89, 7.57)*
Educational level					
No education	1078	2695	2.09 (1.59, 2.75)	1.43 (0.97, 2.11)	1.62 (1.10, 2.39)*
Primary education	1419	4457	1.39 (1.09, 1.79)	1.72 (1.21, 2.43)	1.85 (1.30, 2.62)*
Secondary education	302	1544	0.95 (0.74, 1.24)	1.28 (0.91, 1.18)	1.32 (0.94, 1.87)
Higher education	176	923	1.00	1.00	1.00
Occupation					
Not working	86	858	1.00	1.00	1.00
Professional/technical/managerial	114	517	5.54 (3.74, 8.19)	3.44 (2.10, 5.65)	3.59 (2.18, 5.91)*
Clerical	17	80	7.09 (3.54, 14.19)	8.21 (3.59, 18.77)	8.49(3.68, 19.55)*
Sales	152	525	5.20 (3.55, 7.60)	2.47 (1.58, 3.86)	2.53 (1.61, 3.97)*
Agriculture – employee	2168	6168	4.66 (3.43, 6.34)	2.50 (1.74, 3.59)	2.57 (1.78, 3.72)*
Services	48	162	6.17 (3.68, 10.33)	3.23 (1.74, 5.98)	3.16 (1.70, 5.89)*
Skilled manual	205	619	9.92 (6.88, 14.32)	5.48 (3.57, 8.40)	5.68 (3.68, 8.76)*
Unskilled manual	52	227	5.27 (3.14, 8.83)	3.75(2.02, 6.98)	4.01 (2.14, 7.54)*
Others	132	466	5.11 (3.40, 7.68)	3.69 (2.31, 5.89)	3.70 (2.30, 5.96)*
Religion					
Orthodox	325	5352	1.00	1.00	1.00
Catholic	11	79	0.15 (0.06, 0.38)	0.19 (0.06, 0.60)	0.16 (0.05, 0.48)*
Protestant	98	2648	0.13 (0.09, 0.19)	0.26 (0.17, 0.40)	0.2 (0.13, 0.31)*
Muslin	2505	1411	8.78 (7.04, 10.94)	21.59 (16.00, 9.19)	18.30 (13.54, 4.74)*
Traditional+other	37	129	0.50 (0.06, 0.09)	0.60 (0.20, 1.85)	0.43 (0.14, 1.31)
Sex of household head					
Male	2658	8376	1.56 (1.30, 1.88)	1.01 (0.81, 1.27)	1.03 (0.82, 1.30)
Female	317	1244	1.00	1.00	
Wealth Index					
Poorest	621	1358	1.00	1.00	1.00
Poorer	660	1634	0.64 (0.50, 0.81)	0.71(0.54, 0.93)	0.67(0.51, 0.89)*
Middle	616	1811	0.69(0.54, 0.88)	0.84(0.63, 1.12)	0.80(0.60, 1.08)
Richer	495	2217	0.56(0.43, 0.72)	0.81(0.60, 1.10)	0.76(0.56, 1.03)
Richest	582	2600	0.74(0.55, 1.00)	1.18(0.83, 1.68)	0.94(0.63, 1.39)
Current marital status					
Never in union	854	4036	1.00	1.00	1.00
Married	1814	5154	2.40 (2.09, 2.75)	0.81 (0.63, 1.04)	0.83 (0.64, 1.06)
Living with partner	245	176	2.98 (1.89, 4.70)	1.44 (0.83, 2.50)	1.27 (0.74, 2.19)
Widowed	12	32	4.69 (1.75, 12.58)	1.20 (0.36, 4.020	1.17 (0.34, 3.94)
Divorced	36	189	1.29 (0.76, 2.19)	0.35 (0.19, 0.65)	0.38 (0.21, 0.69)*
No longer living together/separated	13	33	3.17 (1.27, 7.88)	1.86 (0.64, 5.37)	1.88 (0.62, 5.65)
Alcohol drinking in the last 30 days					
Yes	478	4949	1.12(0.95, 1.33)	1.91(1.50, 2.43)	2.15(1.69, 2.73)*
No	2496	4671	1.00	1.00	1.00
Smoking cigarette in the last 30 days					
Yes	526	129	21.62 (16.01, 29.18)	16.87 (12.30, 23.14)	10.79 (8.61,13.51)*
No	2449	9491	1.00	1.00	1.00
Community-level factors					
Residence					
Urban	482	2014	2.13(1.31, 3.45)	1.01(0.58, 1.75)	1.17(0.66, 2.07)
Rural	2493	7606	1.00		
Region					
Oromia	1875	2839	1.00	1.00	1.00
Tigray	19	776	0.01 (0.01,0.03)	0.01 (0.01,0.03)	0.03 (0.01, 0.07)*
Afar	28	54	0.93 (0.35, 2.45)	0.93 (0.35, 2.45)	0.28 (0.11, 0.68)*
Amhara	304	2902	0.04 (0.02, 0.09)	0.04 (0.02, 0.09)	0.07 (0.04, 0.14)*
Somali	142	184	1.60 (0.76, 3.39)	1.60 (0.76, 3.39)	0.36 (0.18, 0.70)*
Benishangul	22	102	0.26 (0.10, 0.68)	0.26 (0.10, 0.68)	0.14 (0.06, 0.35)*
SNNPR	392	2194	0.10 (0.05, 0.21)	0.10 (0.05, 0.21)	0.53 (0.29, 0.99)*
Gambella	8	29	0.30 (0.08, 1.10)	0.30 (0.08, 1.11)	0.78 (0.21, 2.94)
Harari	23	8	11.04 (3.11,39.16)	10.99 (3.02, 40.00)	6.97 (1.91, 25.43)*
Addis Ababa	118	503	0.41 (0.19, 0.87)	0.41(0.17, 0.99)	0.30 (0.14, 0.66)*
Dire Dawa	45	27	4.65 (1.74, 12.37)	4.62 (1.64, 13.02)	2.77 (1.05, 7.30)*

* *P*-value < 0.05

## Discussion

The study determined the prevalence of current khat chewing status and factors affecting it among male adults of Ethiopia. A range of different socio-demographic and economic factors affecting current khat chewing was identified, these include Older age, being uneducated, Muslim religion follower, poorest wealth index, being divorce, history of alcohol drinking for the last 30 days, history of cigarette smoking for the last 1 month, and regions of Harari and Dire Dawa had statistically significant association with khat chewing.

Older people had higher odds of being a khat chewer as compared to teenagers. This finding is in line with other studies conducted in different institutions in Ethiopia [1, 2] and a community-based study on EDHS 2011 [26]. The possible reason could be young people tend to be under family control which reduces their risk of exposure. As a result, their probability of chewing will be less.

Lower educational status was found to be a significant independent predictor of current khat chewing. This finding is in contrast with a study conducted in Butajira, Ethiopia [13]. But, the current study was in agreement with a study conducted from the Jazan region, Saudi Arabia, which showed that illiterates were at higher odds of chewing khat [23]. The reason could be uneducated men would have a lack of information on the negative consequences of khat on their health [27]. Therefore, they would continue khat chewing.

The type of occupation was associated with khat chewing. Accordingly, professionals, clerical, sales, agricultural, service, skilled manual, and unskilled manual workers were at higher odds of being khat chewer. This finding is in line with a study conducted in Ethiopia [26]. This could be due to the fact that jobless individuals cannot afford to buy khat and also they have relatively less work which will stress them so as to need such stimulants.

In this study, Muslims were at higher risk of being khat chewer when compared to Orthodox Christians whereas being Catholic and Protestant was found to decrease the odds of chewing khat. This finding is in line with other studies from Ethiopia [1, 12, 13, 28]. This could be traditionally Muslim religious followers have good acceptance to gain maximum concentration while doing their work and prayer [27, 29].

The odds of khat chewing among poor people were reduced by 33% compared to those with the poorest wealth index. This finding is in agreement with a community-based study from EDHS 2011 [26]. This could be because the poorest people could not even afford basic necessities like food and unable to cover the expense of khat [30].

Divorced men were associated with lower odds of Khat chewing than their single counterparts. This finding is in contrast with a study conducted in Ethiopia [26]. The lower odds of khat chewing among divorced people could be their tendency to modify their lifestyle after divorce as this khat chewing could be also a reason for the divorce. Single individuals usually are also young people who are under family control.

In this study, alcohol drinking is associated with an increased risk of khat chewing as compared to their counterparts. This result is consistent with a systematic review and meta-analysis [31]. A similar association was found from the study conducted in Butajira [13], Saudi Arabia [6], and Uganda [32]. This could be due to the fact that khat chewing causes sleep disturbance [33] as a result, chewers would use alcohol for better sleep. However, different studies showed concurrent use of khat, cigarette, and alcohol would result in sleep deprivation [32, 34, 35]. Therefore, giving due attention is needed to minimize the concurrent effect of khat, alcohol, and cigarette smoking.

Community-level factors of khat chewing were also determined and the region was significantly associated with khat chewing. Men living in the regions of Harari and Dire Dawa were at higher odds of khat chewing. This finding is in line with a study conducted in Ethiopia [26]. This due to the cultural difference across the regions and especially khat chewing in the Eastern part of Ethiopia is culturally acceptable and is considered as a good practice.

This study has a significant implication for the public and policymakers. Khat chewing in the community could affect social life [24], the economy [27], and result in poor health outcomes [35–38]. Currently, in Ethiopia farmers in the rural area are expanding the cultivation of khat and the landmass covered by cereals and fruits is decreasing from time to time. However, the negative effect of khat on health outweighs the income earned from khat production and sale. In addition, khat chewers are prone to poor appetite and resulted in malnutrition. Khat chewing is also a growing concern among Ethiopian universities [39] associated with low academic performance in Ethiopian students [40–42]. Khat chewing contributes to the high burden of non-communicable diseases like cardiovascular disorders [43, 44]. Khat increases the concurrent use of cigarettes and increases risky sexual behavior [9] which further increases the burden of the Human Immune Deficiency Virus (HIV). Khat mainly affects the productive population of a country like university students, youths, and employers this will affect the country’s economy at large in the long run. Therefore, policymakers should design strategies on khat sale and production including high taxation or banning to minimize the burden.

### Limitations

Since the study was cross-sectional it does not show the temporal relationship between the outcome status and the risk factors. Important variables like peer pressure and perceptions/attitude on benefit, consequence, duration of chewing, dosage and frequency were not addressed in this study. So, the long term implications of khat were not studied in this study. However, the authors tried to determine the causes of khat chewing in Ethiopia among the men population.

## Conclusion

Older age, being uneducated, Muslim religion follower, poorest wealth index, being divorce, history of alcohol drinking for the last 30 days, history of cigarette smoking for the last 1 month, and regions of Harari and Dire Dawa had a statistically significant association with khat chewing. Therefore, to effectively control khat chewing among the diverse communities in Ethiopia, policymakers should consider a multi-faceted policy approach that accounts for regional variation, the local social contexts, as well as the complementary nature of smoking and khat chewing practices.

## Data Availability

Data is available on https://dhsprogram.com/data/available-datasets.cfm
